# Comparative metabolomic and physiological analysis uncovers distinct drought tolerance mechanisms in four rice cultivars

**DOI:** 10.1038/s41598-026-41243-6

**Published:** 2026-03-21

**Authors:** Nagy S. Radwan, Sobhi F. Lamlom, Abdul-Hamid Emwas, Mariusz Jaremko, Nader R. Abdelsalam

**Affiliations:** 1 Agricultural Botany Department, Faculty of Agriculture, Alexandria University, Saba Basha, 21531 Alexandria, Egypt; 2 Department of Plant Production, Faculty of Agriculture Saba Basha, Alexandria University, Alexandria, Egypt; 3 Core Lab of NMR, King Abdullah University of Science and Technology (KAUST), Thuwal, 23955-6900 Makkah Saudi Arabia; 4 The Golden Ratio Institute, Riyadh, 13244 Saudi Arabia

**Keywords:** Metabolomics, GC-MS analysis, TCA cycle, Amino acid metabolism, Pathway enrichment, Osmoregulation, Climate resilience, Physiology, Plant sciences

## Abstract

Drought stress is a major abiotic challenge threatening global food security by negatively affecting rice morphology, physiology, and biochemistry. This study explored the morpho-physiological and metabolomic responses of four rice cultivars (Giza 179, Hassawi, Super 300, and Y EGY) to drought stress induced by 15% PEG for 14 days. Measurements included fresh weight, relative water content (RWC), proline content, and plant height, while leaf and root tissues underwent detailed GC-MS metabolomic profiling followed by multivariate analyses such as PCA, VIP scoring, volcano plots, and pathway enrichment. Results showed Y EGY experienced the greatest decline in fresh weight, whereas Giza 179 maintained higher biomass. Stress tolerance indices ranked the cultivars as Giza 179 (85.2%), Super 300 (65.5%), Hassawi (61.7%), and Y EGY (52.2%). Metabolomic analysis identified 114 leaf and 97 root metabolites, with 40 showing VIP > 1.0. Volcano plot analysis revealed distinct variety-specific responses: Giza 179 demonstrated broad metabolic activation with upregulation of methylgalactoside, glycerol-3-phosphate, and citric acid in leaves; Hassawi showed effective targeted responses with strategic trehalose upregulation, while Y EGY exhibited extensive root metabolic disruption. Pathway enrichment identified alanine, aspartate, and glutamate metabolism, arginine and proline metabolism, and the TCA cycle as most significantly affected in leaves, while the TCA cycle was the main response pathway in roots. Post-hoc analysis confirmed that drought-tolerant cultivars exhibited more balanced metabolic regulation. These findings suggest that superior drought tolerance is associated with either efficient targeted metabolic responses that conserve resources (Hassawi, Super 300) or robust integrated mechanisms that coordinate osmoregulation and metabolic reprogramming (Giza 179), providing valuable insights for breeding drought-tolerant rice varieties.

**Supplementary Information:**

The online version contains supplementary material available at 10.1038/s41598-026-41243-6.

## Introduction

Rice (Oryza sativa L.) is an essential staple food grain because half of the world’s population (> 3.5 billion) depends on it for 40–70% of their daily calorie intake^1,2^. Rice contains 80% carbohydrates, 7–8% protein, 3% fat, and 3% dietary fibre, and also contributes nutritionally significant amounts of vitamins like thiamine, riboflavin, niacin, and zinc^3^. Rice is cultivated on over 164 Mha of land in more than 100 countries annually^4^. Boosting rice production is vital to meet the rising global food demand with a projected 9.6 billion population by 2050, as rice is crucial for international and regional food security^5^. To feed the growing population, the 2030 rice production target is 771.02 million tonnes. To meet demand, production must increase by at least 1% annually^6,7^. This poses an enormous challenge: feeding the world while staying within planetary boundaries^8^.

Current studies on global climate change show that it has adversely affected agricultural production and ecological systems in both developed and developing countries^9,10^. Environmental stresses like salinity, drought, cold, higher temperatures, altered rainfall patterns, unpredictable weather, and increasing insect and disease outbreaks harm crop yields and nutritional quality. Among these, drought stands out as a major abiotic stress that greatly hampers cereal production globally^4,11,12^. Recent years have seen a sharp decline in global rice production and grain quality due to climate change and abiotic stresses like drought. Since rice needs much water, drought is a major threat, causing over 90% losses in rice yield^13,14^.. Drought-affected cropping regions may increase fourfold from production to modeling simulations by the end of the twenty-first century^15,16^. The freshwater shortage is a primary cause of low rice yields, as producing 1 kg of rice requires about 3000 L of water. This challenges crop production in over 50% of rice-growing regions. Drought stress causes morphophysiological and biochemical changes in rice, affecting its morphology, yield, and quality across all stages from germination to grain filling^17–19^. Drought stress decreases rice yield by suppressing cell growth, elongation, and expansion. It impairs the plant’s antioxidant defenses by increasing reactive oxygen species levels, leading to oxidative damage of lipids and proteins. This process affects the production of osmotic regulators such as proline, betaine, sorbitol, and mannitol, and disturbs redox balance and ion homeostasis^17,20^. Stomata closure reduces CO2 inflow, thereby lowering photosynthetic activity, which in turn reduces metabolic activity in the plant^21^.

Metabolomics is now regarded as a comprehensive, sensitive, and practical method for obtaining valuable insights into the composition of metabolite pools across various organisms, including plants^22,23^. Plant metabolomics has emerged as a powerful method for investigating various aspects of system biology, significantly enhancing our understanding of metabolic and signaling pathways involved in plant growth, development, and stress responses, with the aim of improving crop quality and yield^24^. Various innovative techniques like mass spectrometry, gas chromatography-mass spectrometry (GC-MS), liquid chromatography-mass spectrometry (LC-MS), capillary electrophoresis-mass spectrometry (CE-MS), Fourier transform ion cyclotron resonance-mass spectrometry (FTICR-MS), matrix-assisted laser desorption/ionization (MALDI), ion mobility spectrometry (IMS), and nuclear magnetic resonance (NMR) are all wonderful tools used to study large, complex mixtures of plant extracts^25^. The application of GC–MS to profile stress-responsive metabolites, which help plants adapt to adverse conditions, is crucial in contemporary biotechnology research^26–28^.

Recently, interest in how plants’ metabolism responds to drought stress has grown, as many metabolites are thought to play crucial roles in stress tolerance. Targeting a metabolite is more effective for improving drought tolerance than focusing on a single gene, since metabolites are produced through interactions among multiple genes and pathways, resulting in broader effects on stress responses^29–31^. The metabolic profile of drought-stressed barley and maize leaves and roots showed significant accumulation of metabolites involved in glyoxylate and dicarboxylate metabolism in maize roots and isoflavonoid biosynthesis in barley roots^32–34^. Prolonged water deficiency in plants led to a noticeable increase in amino acids, tricarboxylic acid cycle (TCA) metabolites, and secondary metabolites in Pinus sylvestris leaves^35^.

In this study, we used GC-MS to analyze the metabolic profiles of leaves and roots from four rice varieties (Giza 179, Super 300, Y EGY, and Hassawi rice) under both control and drought conditions. We also examined their morphological and physiological traits simultaneously. The four varieties showed notable differences in how their metabolisms responded to drought stress. Our results provide insight into the specific ways rice adapts to drought conditions.

## Materials and methods

### Plant materials and growth conditions

An experiment was carried out during the summer of 2023. Three Oryza sativa cultivars were sourced from the Rice Research Department in Sakha, Kafr El-Sheikh, Egypt, while the fourth came from King Abdullah University of Science and Technology. These cultivars are among the most widely cultivated in Egypt and Saudi Arabia because of their high yield, morphological characteristics, and adaptability to climate variability. The study focused on assessing drought stress effects on these cultivars at the seedling stage. Seeds were surface sterilized with 3% sodium hypochlorite and germinated on moist blotting paper at approximately 25 ± 2 °C. Once pre-germinated, seeds from each genotype were planted in plastic trays (30 cm × 20 cm × 10 cm) filled with a 3:1 mixture of field soil and farmyard manure. Post-germination, the seedlings were transferred to a growth chamber set to 70% humidity, 24 ± 2 °C, with a 16-hour photoperiod and a photosynthetic photon flux density of 250–350 µmol m − 2 s − 1.

### Drought stress treatment application and experimental design

Germination rates were recorded after 96 h of incubation. Seeds were deemed germinated if the root length was at least 1 cm and the shoot length was at least 0.5 cm. Initial testing with various PEG6000 concentrations (5%, 10%, 15%, and 20%) indicated that 15% PEG was the most effective for assessing drought tolerance among the cultivars, and it was therefore selected for the drought treatment. Polyethylene glycol (PEG-6000) was selected over water withholding for several scientific reasons. PEG induces uniform, reproducible osmotic stress across all plants simultaneously, eliminating spatial heterogeneity inherent in soil-drying experiments^36^; PEG-mediated stress is physiologically equivalent to drought as it creates an osmotic potential gradient without causing nutrient imbalances; PEG allows precise control of stress intensity through concentration adjustment; PEG-induced osmotic stress at the seedling stage is a well-established method for screening drought tolerance in crops^36^; and the controlled nature of PEG treatment enables direct comparison of metabolomic responses across cultivars without confounding environmental variables present in water withholding experiments. This approach is particularly well-suited for metabolomic studies, where reproducibility and standardization are critical.

The experiment employed a randomized complete block design (RCBD) with four replicates. Cultivars (A) included Giza 179, Super 300, Y EGY, and Hassawi, while abiotic treatments (B) consisted of a control without stress and drought stress with 15% PEG applied for 14 days. Each treatment was replicated four times, with each replicate containing 20 seeds. For drought stress induction, 15% (w/v) PEG-6000 solution was prepared by dissolving 150 g of PEG-6000 (molecular weight 6000 Da) in 1 L of distilled water and stirring until complete dissolution. The solution was applied to 14-day-old seedlings by replacing the irrigation water with PEG solution. Each pot received 200 mL of 15% PEG solution, which was maintained at constant volume throughout the 14-day treatment period by adding PEG solution as needed to compensate for evaporation. Control plants received equivalent volumes of distilled water. The PEG solution was prepared fresh every 3 days to maintain consistent osmotic potential and prevent microbial contamination.

### Growth characters

#### Stress tolerance index calculation

To compare overall stress performance independent of baseline vigor differences, we calculated a drought tolerance index as the mean percentage retention of six growth parameters under PEG relative to control conditions. For each cultivar, the following parameters were measured under both control (CK) and drought stress (15% PEG) conditions: shoot fresh weight (FW), shoot dry weight (DW), root fresh weight (RFW), root dry weight (RDW), chlorophyll content (CH), and plant height (PH). The stress tolerance index (STI) was then computed as:

STI (%) = [(FW_PEG/FW_CK + DW_PEG/DW_CK + RFW_PEG/RFW_CK + RDW_PEG/RDW_CK + CH_PEG/CH_CK + PH_PEG/PH_CK)/6] × 100.

This integrated index provides a comprehensive measure of drought tolerance by capturing multiple dimensions of plant stress response across both shoot and root systems, as well as photosynthetic capacity. Values above 100% indicate superior drought tolerance with parameter maintenance or enhancement under stress conditions, while values below 100% reflect stress-induced performance reduction. By averaging across multiple growth and physiological parameters, this index normalizes for cultivar-specific baseline differences and provides a robust comparison of relative stress tolerance independent of absolute plant size or vigor.

#### Relative water content

RWC was estimated from two biological replications following a 14-day period of stress imposition, with fresh weight recorded immediately thereafter. A sample comprising 10–15 leaf discs, each 1 cm in diameter, was prepared to float in Petri dishes containing distilled water at room temperature for 4 h. The turgid weight (TW, g) of each leaf disc was subsequently recorded after removing excess water with tissue paper. The leaf discs were then enclosed in butter paper bags and dried in a hot air oven (Sisco, India Pvt. Ltd.) set at 85 °C for 24 h. The dry weight (DW, g) of the samples was subsequently measured and calculated using the appropriate method:

*RWC (%) = [(FW - DW)/(TW - DW)] × 100*.

where FW = fresh weight, DW = dry weight, TW = turgid weight.

### Chlorophyll content

Total chlorophyll index measures the green color intensity (SPAD units), assessed using a chlorophyll meter (SPAD-502, Minolta Co., Japan). It is represented by the average SPAD value from ten randomly selected leaves in each subplot at 90 DAS, following the method described by^37^.

### Biochemical analyses

#### Proline content determination

Proline content was quantified using a modified ninhydrin-based colorimetric method^38^. Fresh leaf tissue (100 mg) was ground in 3% (w/v) sulfosalicylic acid (5 µL per mg of fresh weight) and centrifuged at 12,000× g for 5 min at 4 °C. The supernatant (100 µL) was then combined with an equal volume of reaction mixture containing 3% sulfosalicylic acid, glacial acetic acid, and acidic ninhydrin (1:2:2, v/v/v). This mixture was heated at 96 °C for 60 min, then extracted with 1 mL of toluene. After phase separation, the toluene layer containing the chromophore was collected, and absorbance was read at 520 nm using a NanoDrop 2000 spectrophotometer (Thermo Scientific, Wilmington, DE, USA)^39^. Proline levels were determined through a standard curve and reported as µmol per gram of fresh weight, using this formula:

Proline (µmol g⁻¹ FW) = [(µg proline mL⁻¹ × mL toluene)/115.5]/(g FW/5)^40^.

#### Metabolite extraction and GC-MS analysis

Metabolite extraction followed a methanol-based method^23^. Specifically, 100 mg of leaf tissue ground in liquid nitrogen was extracted with 500 µL of 80% (v/v) methanol, vortexed, and kept on ice for 5 min. After centrifuging at 12,000× g at 4 °C for 20 min, the supernatant was diluted with ultrapure water to reach 53% methanol content. The final supernatant underwent a second centrifugation before GC-MS analysis. Chromatography was carried out on a Hypersil Gold C18 column at 40 °C, with a flow rate of 0.2 mL/min, using mobile phases of 0.1% formic acid in water (A) and methanol (B) at pH 9.0. UHPLC-MS/MS analyses utilized a Vanquish UHPLC system (Thermo Fisher Scientific, Bremen, Germany).

### Statistical analysis

#### Metabolomic data preprocessing and quality control

Raw GC-MS data preprocessing involved multiple quality control steps to ensure data reliability. Peak intensity filtering was performed to remove low-abundance signals and potential artifacts. Specifically, metabolites were retained for analysis if they showed peak intensity > 1000 arbitrary units (AU) in at least 75% of samples within at least one treatment group (control or PEG stress). This threshold-based approach ensures that included metabolites represent consistently detectable compounds rather than noise or sporadic signals, while the 75% criterion allows for biological variation within treatment groups without excluding genuine stress-responsive metabolites that may be present at low levels in unstressed plants. Missing values, which occasionally arise from peaks falling below detection limits or integration failures, were imputed using the k-nearest neighbors (k-NN) algorithm with k = 5. This method estimates missing values based on the five most similar samples in multivariate space, preserving the correlation structure of the data more effectively than simple mean imputation or zero-filling approaches. Quality control samples were analyzed at regular intervals throughout the GC-MS runs to monitor instrumental stability and ensure data consistency across batches.

Prior to multivariate statistical analyses, metabolite abundance data were log-transformed (base 2) to approximate normal distribution and reduce heteroscedasticity, a common characteristic of metabolomics datasets where variance often scales with mean abundance. Subsequently, data were Pareto-scaled, which divides each variable by the square root of its standard deviation. Pareto scaling reduces the influence of high-abundance metabolites while preserving the data structure better than unit variance (UV) scaling, making it particularly suitable for metabolomics data where both high- and low-abundance compounds may be biologically important. This preprocessing pipeline ensures equal weighting of metabolites with different concentration ranges during multivariate modeling, preventing dominant metabolites from obscuring subtle but meaningful changes in less abundant compounds.

#### Univariate and multivariate statistical analyses

Statistical analyses were conducted utilizing MetaboAnalyst 5.0 (https://www.metaboanalyst.ca/, accessed on 20-3-2025). The dataset was subjected to both univariate and multivariate statistical analyses. For univariate comparisons between control and drought-stressed samples within each cultivar, fold-change analysis and Student’s t-tests were performed, with significance thresholds set at |log2FC| > 1 and *p* < 0.05. These dual criteria ensure that identified metabolites show both a substantial magnitude of change (> 2-fold) and statistical reliability. Multivariate analyses included hierarchical cluster analysis (HCA) using Euclidean distance and Ward’s linkage method to visualize metabolite grouping patterns; principal component analysis (PCA) to explore overall variance structure and identify clustering trends without supervision; and partial least squares discriminant analysis (PLS-DA) to maximize separation between treatment groups while identifying metabolites contributing to class discrimination. PLS-DA model validity was assessed through cross-validation (7-fold) and permutation testing (*n* = 1000) to prevent overfitting. Variable importance in projection (VIP) scores were calculated from PLS-DA models to identify metabolites with the highest discriminatory capacity, with VIP > 1.0 indicating substantial importance and VIP > 1.5 denoting high discriminatory power.

Statistical significance for differential metabolite abundance was established at *p* < 0.05. For multiple comparison correction in large-scale metabolite screening, false discovery rate (FDR) adjustment using the Benjamini-Hochberg method was applied where appropriate. Morpho-physiological data (fresh weight, dry weight, RWC, chlorophyll content, plant height) were analyzed using two-way analysis of variance (ANOVA) with cultivar and treatment as fixed factors, followed by Tukey’s HSD post-hoc test for pairwise comparisons (α = 0.05). All experimental procedures were performed in accordance with institutional guidelines and regulations.

## Results

### Effects of PEG-Induced drought stress on growth and physiological parameters

**Fig. 1:**
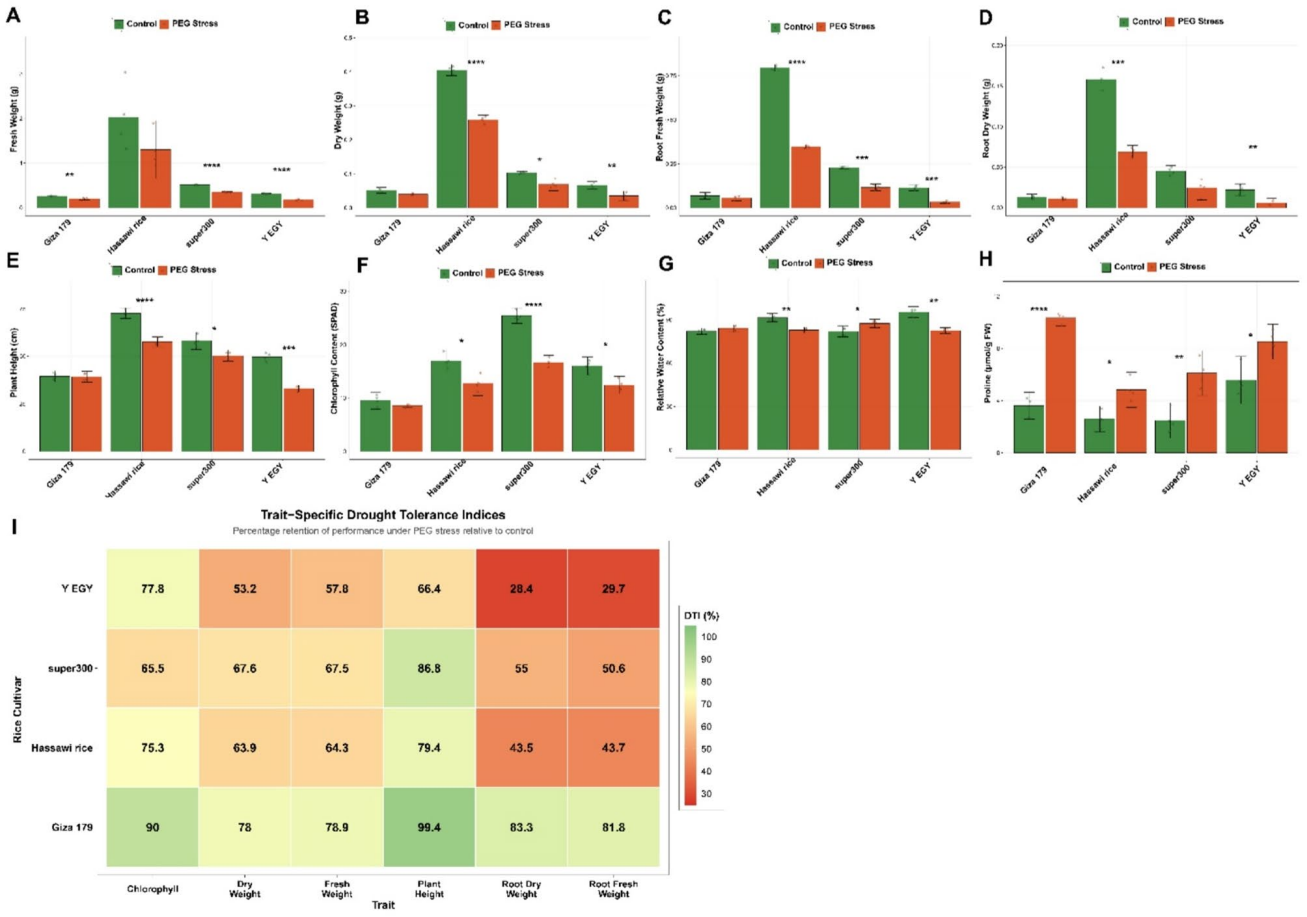
Effects of PEG-induced drought stress on growth and physiological parameters in four rice cultivars. (**A**) Fresh weight, (**B**) Dry weight, (**C**) Root fresh weight, (**D**) Root dry weight, (**E**) Plant height, (**F**) Chlorophyll content, (**G**) Relative water content, (**H**) Proline content. (**I**) Drought tolerance index calculated as the average percentage retention of six growth parameters under stress conditions. Bars represent means ± SE (*n* = 3–5 biological replicates). Asterisks indicate significant differences between control (CK) and PEG treatments within each cultivar (* *P* < 0.05, ** *P* < 0.01, *** *P* < 0.001; Bonferroni-adjusted *t*-test). The dashed red line in panel I indicates 100% performance (no stress effect).

Analysis of variance revealed that cultivar identity, PEG treatment, and their interaction exerted highly significant effects on all measured parameters. Hassawi rice produced substantially greater biomass than other cultivars under both control and stress conditions. Under well-watered conditions, this genotype accumulated 2.02 g shoot fresh weight and 0.405 g dry weight (Fig. 1A-B). PEG treatment reduced fresh weight to 1.30 g (36% reduction) and dry weight to 0.259 g (36% reduction), though absolute values remained well above those of other genotypes. The cultivar super300 showed intermediate performance, declining from 0.515 g to 0.348 g fresh weight (32% reduction) under stress, while Giza 179 and Y EGY exhibited smaller absolute biomass but proportionally similar stress-induced reductions.

Root biomass followed parallel trends, with Hassawi rice maintaining the highest values under both treatments (Fig. 1C-D). Root fresh weight declined from 0.796 g to 0.348 g (56% reduction), while root dry weight decreased from 0.158 g to 0.069 g (56% reduction). These proportional reductions in root biomass exceeded those observed for shoot biomass across all cultivars, suggesting that PEG-mediated osmotic stress may preferentially constrain root development or that cultivar adaptation strategies involve differential allocation of limited resources between root and shoot systems. Plant height differed substantially among cultivars, with Hassawi rice reaching 72.6 cm under control conditions compared to other genotypes (Fig. 1E). PEG treatment reduced height across all cultivars, with Hassawi rice showing the largest absolute decrease (21% reduction to 57.6 cm) and Y EGY the most pronounced proportional effect (34% reduction to 33.0 cm). These architectural responses likely integrate both direct effects of osmotic stress on cell expansion and indirect consequences of altered resource allocation.

Chlorophyll content varied markedly among cultivars under control conditions, ranging from 9.5 units in Giza 179 to 25.4 units in Super 300 (Fig. 1F). PEG treatment reduced chlorophyll across all genotypes, though the magnitude of decline differed. The cultivar Super 300 experienced the largest absolute decrease (from 25.4 to 16.7 units, 35% reduction), while Giza 179 showed the smallest proportional change (10% reduction). These differential responses likely reflect variation in both baseline pigment concentrations and stress-protection mechanisms. Relative water content exhibited complex cultivar-dependent patterns (Fig. 1G). Under control conditions, Y EGY maintained the highest RWC at 95.3%, followed by Hassawi rice (91.5%), Super 300 (81.9%), and Giza 179 (82.1%). Osmotic stress induced divergent responses: while most cultivars showed modest changes, super 300 paradoxically increased RWC from 81.9% to 87.4% under PEG treatment, potentially reflecting enhanced osmotic adjustment capacity. Proline concentration increased substantially in all cultivars following PEG exposure, consistent with its well-established role as an osmoprotectant (Fig. 1H). The magnitude of induction varied among genotypes: Giza 179 showed the strongest response (2.9-fold increase from 3.6 to 10.4 mol/g FW), followed by super300 (2.5-fold), Hassawi rice (1.9-fold), and Y EGY (1.5-fold). Notably, Y EGY maintained elevated baseline proline under control conditions (5.6 mol/g), suggesting constitutive expression of stress-preparedness mechanisms. The inverse relationship between baseline proline levels and stress-induced fold-changes across cultivars may reflect different regulatory strategies or metabolic constraints on total proline accumulation.

To compare overall stress performance independent of baseline vigor differences, we calculated a drought tolerance index as the mean percentage retention of six growth parameters under PEG relative to control conditions (Fig. 1I). This metric revealed Giza 179 as the most stress-tolerant genotype (DTI = 85.2%), maintaining function across traits despite its modest absolute size. The cultivar super300 ranked second (DTI = 65.5%), followed by Hassawi rice (61.7%) and Y EGY (52.2%). Examination of trait-specific tolerance indices provided additional insight into stress response mechanisms. Giza 179 exhibited remarkably consistent tolerance across parameters, with retention values ranging from 78.0% (FW and DW) to 99.4% (PH). In contrast, Hassawi rice and Y EGY showed pronounced trait-specific vulnerabilities, particularly in root biomass parameters. These patterns suggest that high-biomass cultivars may incur proportionally greater stress penalties, possibly due to higher resource demands or architectural constraints under water limitation.

The dissociation between absolute performance and proportional stress tolerance observed here highlights a fundamental challenge in breeding for drought resistance. While Hassawi rice dominated under both control and moderate stress conditions in absolute terms, Giza 179 proved superior in maintaining functional homeostasis when normalized for baseline differences. This finding suggests that optimal cultivar selection depends critically on target environment characteristics: high-yielding genotypes with moderate tolerance may excel under intermittent stress, whereas low-vigor but highly tolerant types could prove more reliable under severe or sustained drought.

### Multivariate analysis of GC-MS metabolomic data reveals tissue-specific and cultivar-dependent responses

**Fig. 2:**
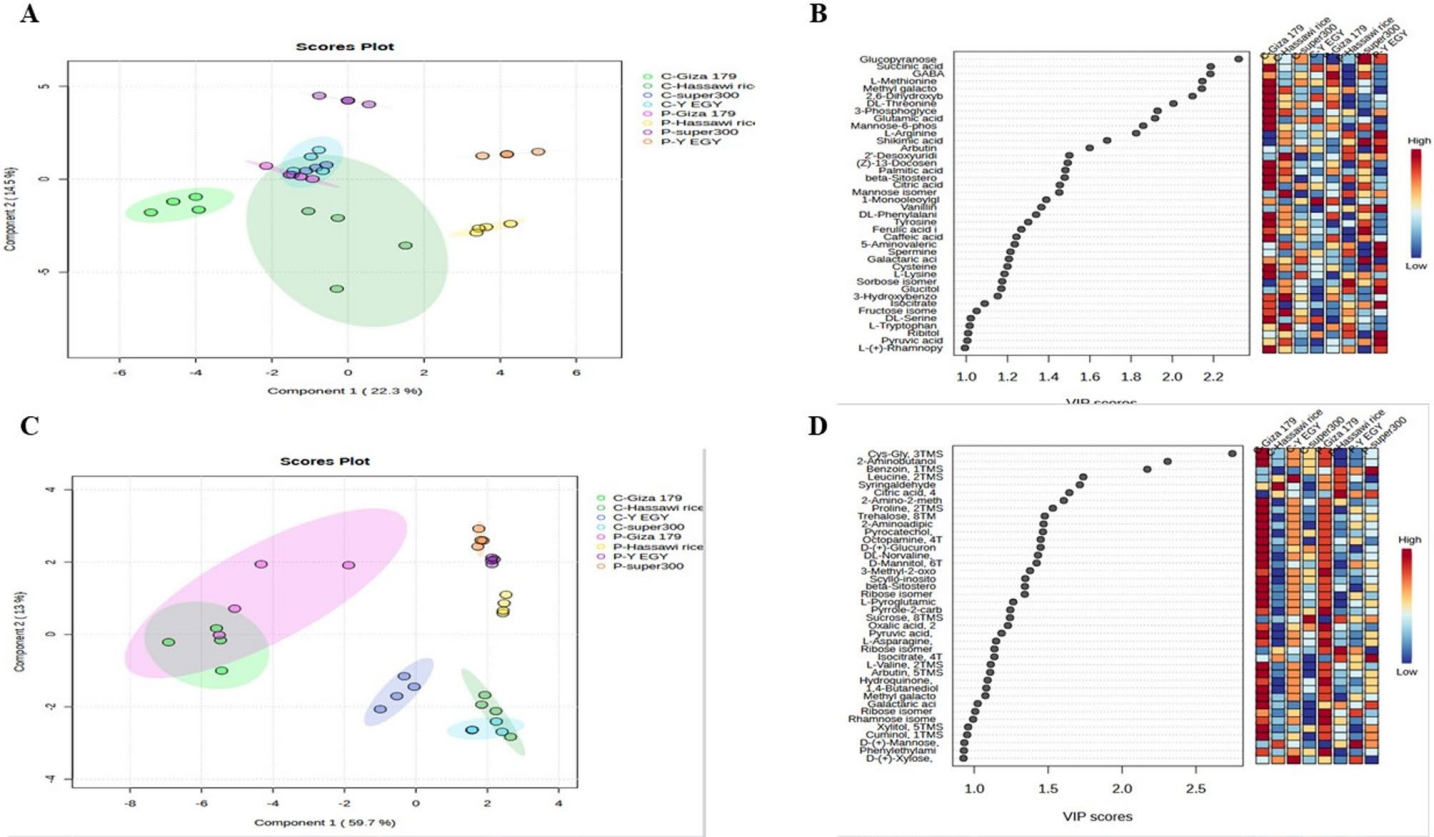
Principal Component Analysis (PCA) and Variable Importance in Projection (VIP) analysis of metabolomic profiles from four rice cultivars under control and drought tress conditions based on GC-MS data. (**A**) PCA score plot of leaf tissue metabolites with PC1 explaining 28.4% and PC2 accounting for 16.1% of total variance. (**B**) VIP score plot for leaf samples, illustrating metabolite importance rankings based on weighted sums of squares of PLS loadings and explained variance contributions. (**C**) PCA score plot of root tissue metabolites with PC1 explaining 65.9% and PC2 accounting for 10.4% of total variance. (**D**) VIP score plot for root samples, displaying the relative importance of individual metabolites in distinguishing treatment groups and cultivar responses.

Principal component analysis was performed on GC-MS metabolomic data to assess variations in metabolite profiles among rice cultivars under control and drought stress conditions. PCA score plots were generated separately for leaf tissue (Fig. 2A) and root tissue (Fig. 2C) samples to illustrate clustering patterns and treatment-induced metabolomic changes. Leaf tissue metabolism analysis showed moderate differentiation between drought-stressed and control samples, with the first two principal components accounting for 44.5% of the total variance (PC1: 28.4%, PC2: 16.1%). Clustering patterns showed overlap among all cultivars under both control and drought-stressed conditions. These overlapping patterns suggest underlying metabolic characteristics shared by specific cultivars and the potential for metabolic affinity induced by stress. Root tissue analysis revealed more pronounced segregation patterns, with the first two principal components accounting for 76.3% of the total variance (PC1: 65.9%, PC2: 10.4%). Metabolic differentiation was evident for Giza 179 under both control and drought conditions, while both Super 300 cultivars under drought conditions overlapped with Y EGY under control conditions. This tissue-specific response pattern suggests that root metabolic profiles may be more sensitive to cultivar-specific drought stress adaptations than leaf tissues. Principal component analysis models demonstrated sufficient explanatory power, with each tissue type representing more than 60% of the metabolic variance, thus validating the effectiveness of the multivariate analysis approach. The statistical significance of metabolite loads was validated using Kruskal-Wallis ANOVA with false discovery rate (FDR) correction at α = 0.05. VIP scores serve as quantitative measures of metabolite significance within the analytical framework. Values exceeding 1.0 indicate substantial importance, while scores below 1.0 suggest reduced relevance to the experimental design. The metabolomic analysis identified 40 metabolites with VIP scores > 1.0 in both leaf tissues (Fig. 2B) and root systems (Fig. 2D), established through GC-MS profiling. These metabolites significantly contributed to the phenotypic variations observed among cultivars under salinity stress. A threshold of VIP > 1.5 was applied to identify metabolites with optimal discriminatory capacity, resulting in the selection of 1 0significant metabolites that contributed to notable inter-group variance. This subset represents the primary biochemical factors underlying the differential stress responses observed among the four cultivars. The complete metabolomic dataset comprised 114 polar metabolites from leaf tissues and 97 from root systems, demonstrating the substantial metabolic complexity captured through GC-MS analysis.

### Hierarchical clustering and comparative analysis

**Fig. 3:**
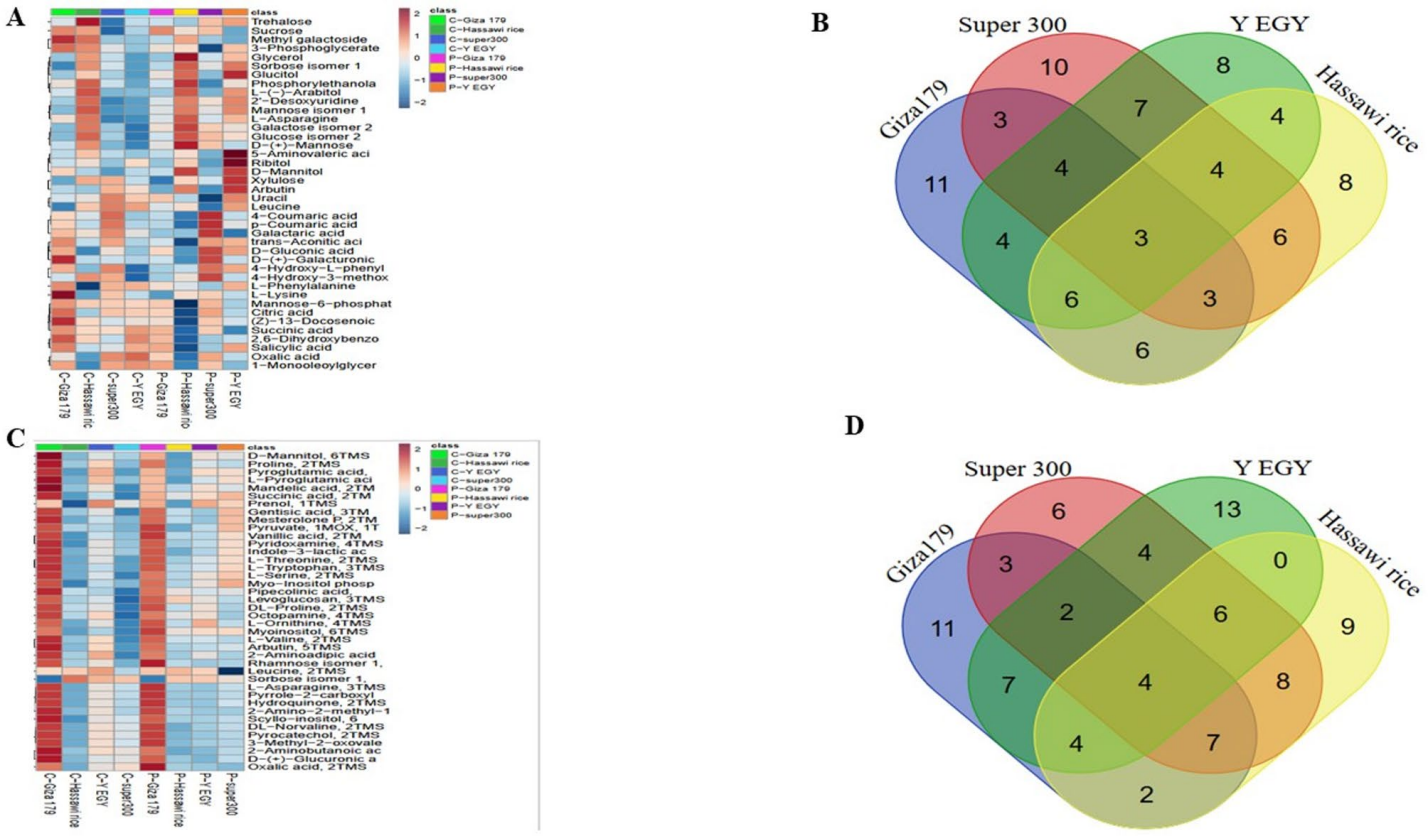
Metabolomic profiling and comparative analysis of drought stress responses across four rice cultivars. Heatmaps display hierarchical clustering of the top 40 most variable metabolites based on VIP scores, with experimental conditions arranged in rows and metabolite profiles in columns. Color intensity represents relative metabolite concentrations, where red indicates elevated abundance and blue denotes reduced levels. (**A**) Leaf tissue metabolomic heatmap analysis. (**B**) Venn diagram illustrating shared and unique metabolites among cultivars at the leaf level. (**C**) Root tissue metabolomic heatmap analysis. (**D**) Venn diagram showing metabolite distribution patterns among cultivars at the root level.

Hierarchical clustering analysis of metabolomic profiles revealed clear tissue-specific responses to drought stress at both foliar and root levels. Heatmap visualization used a matrix layout with rows representing experimental conditions (control versus drought-treated samples) and columns showing individual metabolite profiles. Metabolite concentrations were depicted using a color-coded intensity scale, where red indicates high abundance and blue indicates lower concentrations relative to the dataset. A detailed heatmap analysis of the top 40 VIP-selected metabolites provided a clear view of concentration changes across treatment conditions. Leaf-level responses are shown in Fig. 3A, while root-level patterns are displayed in Fig. 3C. This analysis identified tissue-specific and cultivar-dependent metabolic reprogramming in response to drought stress, highlighting the complex mechanisms behind plant stress adaptation. Venn diagram analysis was used to identify both shared and unique metabolomic signatures across the four rice cultivars, allowing for the characterization of cultivar-specific stress responses. Among the 114 metabolites identified in leaf tissues, four showed consistent expression patterns across all four cultivars (Fig. 3B), indicating conserved metabolic pathways linked to foliar drought tolerance. Root metabolomic analysis of 97 identified compounds revealed that only three metabolites displayed universal expression patterns across cultivars (Fig. 3D), suggesting greater metabolic diversity in root tissue responses to osmotic stress.

### Volcano plot analysis of differential metabolite expression under drought stress

Analysis of the volcano plots revealed distinct, variety-specific metabolic responses to drought stress, with each rice variety exhibiting unique patterns in the regulation of its metabolites across both leaf and root tissues. Throughout this analysis, only metabolites meeting both significance thresholds (|log2FC| > 1 and *p* < 0.05) are discussed as significantly altered or differentially expressed.

**Fig. 4:**
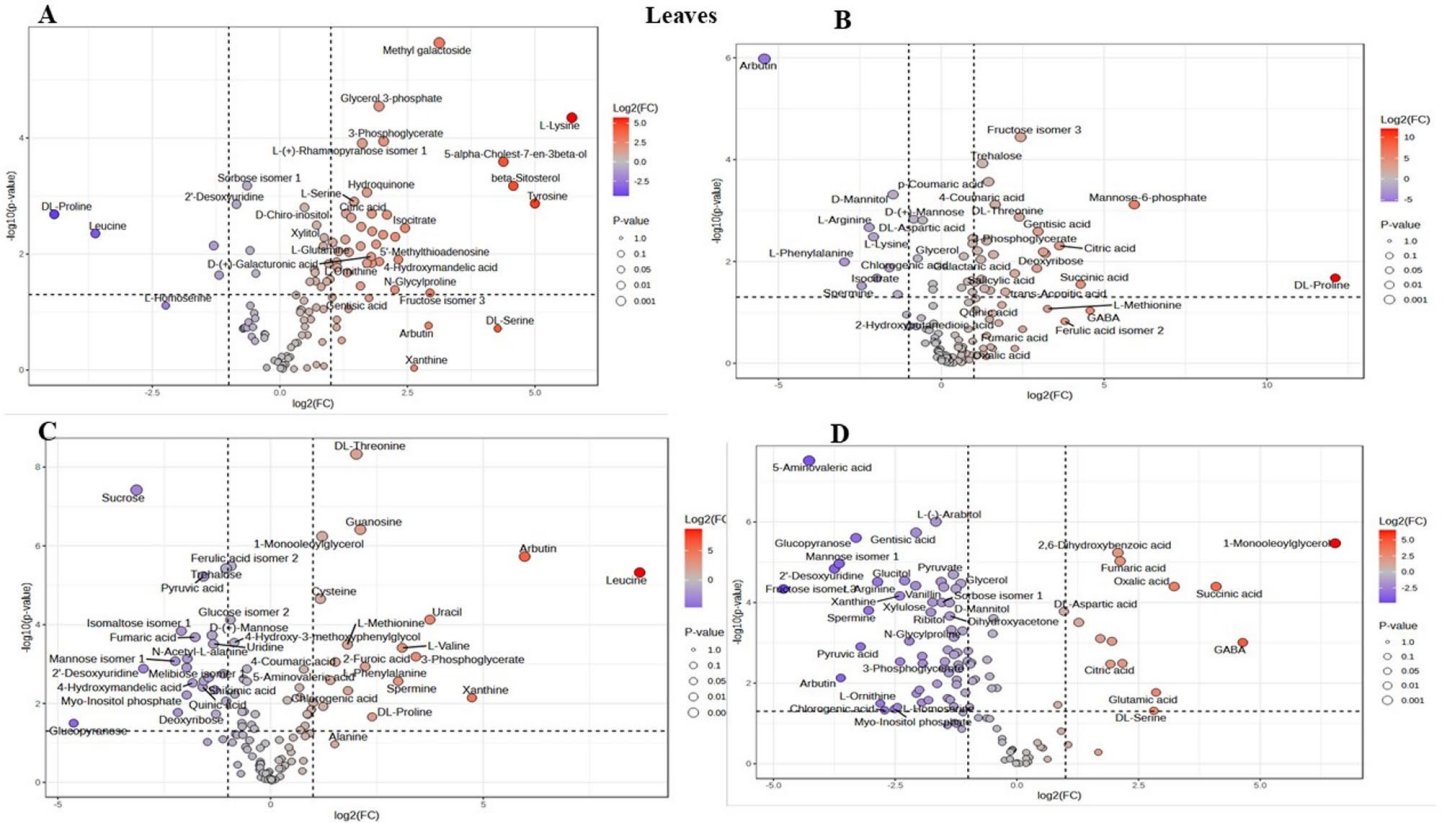
Leaf tissue metabolomic responses to drought stress across rice cultivars. Volcano plots display differential metabolite accumulation in Leaf tissues. (**A**) Giza 179, (**B**) Y EGY, (**C**) Super 300, and (**D**) Hassawi rice following 14-day exposure to 15% PEG. The X-axis shows log2 (fold change) relative to control conditions, and the Y-axis shows -log10(p-value) for statistical significance. Color gradient represents fold-change magnitude: red indicates upregulation, blue indicates downregulation. Metabolite names are labeled for compounds exceeding significance thresholds (|log2FC| > 1, *p* < 0.05). Point sizes correspond to p-value significance levels.

In leaf tissues (Fig. 4A), the Giza 179 variety showed the most pronounced metabolic disturbance under drought conditions, with marked upregulation of several metabolites meeting significance criteria (|log2FC| > 1, *p* < 0.05) including amino acids, organic acids, and sugars such as methylgalactoside, glycerol-3-phosphate, L-lysine, 3-phosphoglycerate, and citric acid, with several showing particularly high fold changes (log2FC > 2), indicating broad activation of stress response pathways with high statistical significance (-log10(p-value) > 2). Conversely, several metabolites, including DL-proline, leucine, and 2’-deoxyuridine, were markedly reduced (among those meeting significance criteria), suggesting metabolic redistribution under stress conditions. This aggressive, broad-spectrum response suggests an energetically costly adaptation mechanism. The Hassawi rice cultivar (Fig. 4B) exhibited a remarkably restrained metabolic response with fewer significantly altered metabolites and generally lower fold variations compared to other cultivars among those exceeding thresholds, yet demonstrated strategic upregulation of key stress-protective metabolites, most notably trehalose, which emerged as a highly regulated compound (|log2FC| > 1, *p* < 0.05) indicating activation of osmotic modulation mechanisms. Other significantly regulated compounds in Hassawi included fructose isomer 3, mannose-6-phosphate, succinic citric acid, and gentisic acid, while compounds such as DL-aspartic acid, L-phenylalanine, and L-lysine showed reduced regulation. This relatively modest metabolic disturbance, coupled with targeted upregulation of critical osmoprotectants, suggests a more efficient and precise stress response mechanism that conserves resources while maintaining protection, contributing to Hassawi’s superior drought tolerance performance. The Super 300 cultivar (Fig. 4C) exhibited a moderate but robust metabolic response in leaf tissues, with marked increases in amino acids meeting significance thresholds including DL-threonine, leucine, and L-methionine, along with increased production of phenolic glycosides such as arbutin, while sucrose, ferulic acid isomer 2, trehalose, pyruvic acid, mannose isomer 1, and fumaric acid decreased (among significant metabolites), indicating selective activation of specific metabolic pathways. The Y EGY cultivar (Fig. 4D) exhibited a distinct metabolic pattern characterized by fewer metabolites meeting significance thresholds and generally lower fold changes among those that did, showing elevated levels of organic acids including succinic acid, oxalic acid, and fumaric acid, along with amino acids such as DL-aspartic acid and GABA (among significant metabolites), while levels of compounds such as 5-aminovaleric acid, mannose isomer 1, and arbutin were reduced. This overall pattern suggests a more controlled adaptation strategy compared to the more dramatic responses observed in Giza 179.

**Fig. 5:**
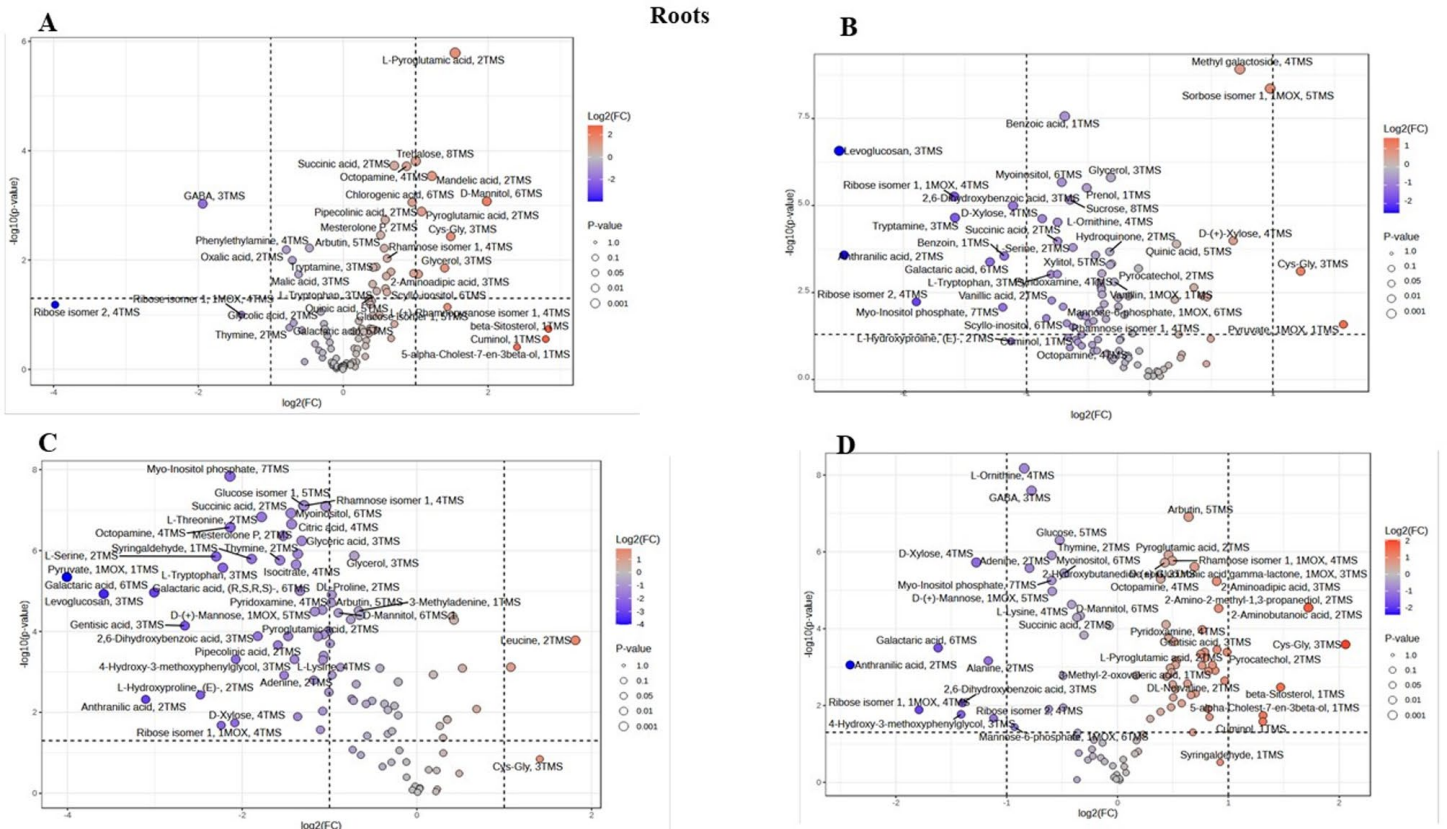
Root tissue metabolomic responses to drought stress across rice cultivars. Volcano plots displaying differential metabolite accumulation in root tissues of tissues (**A**) Giza 179, (**B**) Y EGY, (**C**) Super 300, and (**D**) Hassawi rice following 14-day exposure to 15% PEG. The X-axis shows log2(fold change) relative to control conditions, and the Y-axis shows -log10(p-value) for statistical significance. Color gradient represents fold-change magnitude: red indicates upregulation, blue indicates downregulation. Metabolite names are labeled for compounds exceeding significance thresholds (|log2FC| > 1, *p* < 0.05). Point sizes correspond to p-value significance levels.

In root tissues (Fig. 5A), Giza 179 again demonstrated broad metabolic activation with marked upregulation of L-pyroglutamic acid 2TMS, octopamine 4TMS, succinic acid 2TMS, mandelic acid 2TMS, and trehalose 8TMS (all exceeding |log2FC| > 1, *p* < 0.05), while several metabolites, including GABA 3TMS, ribose isomer 2 4TMS, phenylethylamine 4TMS, and oxalic acid 2TMS were significantly downregulated, indicating comprehensive metabolic redistribution under stress conditions. Hassawi rice (Fig. 5B) exhibited a moderate yet robust root metabolic response with significant increases (exceeding |log2FC| > 1, *p* < 0.05) in methyl galactoside 4TMS, sorbose isomer 1 1MOX 5TMS, S-Cys-Gly 3TMS, and quinic acid 5TMS, indicating selective activation of specific metabolic pathways, while compounds such as levoglucosan 3TMS, tryptamine 3TMS, benzoin 1TMS, and succinic acid 2TMS were reduced. Interestingly, Super 300 (Fig. 5C) exhibited the most restrained root metabolic response among metabolites meeting significance criteria (|log2FC| > 1, *p* < 0.05), with fewer significantly altered metabolites and generally lower fold variations among those exceeding thresholds compared to other varieties, a pattern opposite to its leaf tissue response. Leucine 2TMS and S-Cys-Gly 3TMS emerged as highly regulated metabolic products in Super 300 roots (meeting significance criteria), while compounds such as glucose isomer 1 5TMS, succinic acid 2TMS, L-threonine 2TMS, L-serine 2TMS, and L-tryptophan 3TMS showed reduced regulation. This relatively modest root metabolic disturbance in Super 300, coupled with strategic regulation of key stress-protective metabolites, mirrors the efficient response strategy observed in Hassawi leaf tissues and suggests a more targeted stress response mechanism contributing to drought tolerance. The Y EGY variety (Fig. 5D) exhibited the most pronounced root metabolic perturbation under drought treatment, with notable upregulation of amino acids meeting significance thresholds such as Cys-Gly 3TMS, pyroglutamic acid 2TMS, and 2-aminoadipic acid 3TMS, along with sugar-related metabolites including rhamnose isomer 1 1MOX 4TMS and D-(+)-glucuronic acid gamma-lactone 1MOX 3TMS, indicating broad activation of stress-responsive pathways. Conversely, several metabolites, including L-ornithine 4TMS, GABA 3TMS, galactaric acid 6TMS, and D-xylose 4TMS, were significantly downregulated (all exceeding significance thresholds), suggesting extensive metabolic reallocation under stress conditions. These contrasting strategies across varieties and tissues—with Hassawi and Super 300 demonstrating efficient, targeted responses versus Giza 179 and Y EGY showing broad metabolic activation—likely reflect different evolutionary adaptations to water stress and provide valuable insights for breeding drought-tolerant rice varieties.

### Differential accumulation of key discriminatory metabolites across rice cultivars

**Fig. 6:**
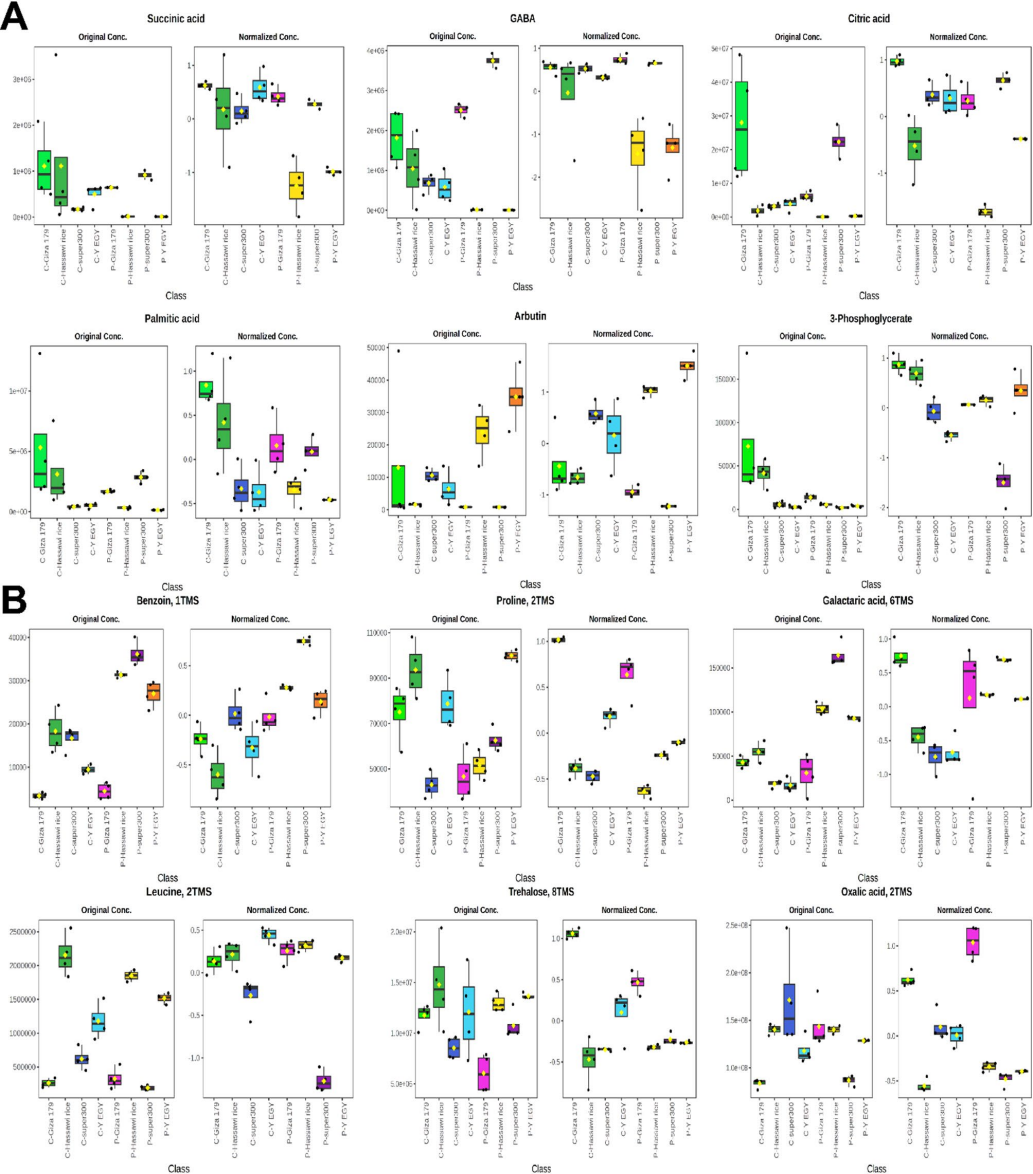
Box plot analysis of key metabolites distinguishing rice cultivars under control and drought stress conditions. (**A**) Leaf tissue metabolite profiles showing raw concentrations (left panels) and normalized data (right panels) for succinic acid, GABA (γ-aminobutyric acid), citric acid, palmitic acid, arbutin, and 3-phosphoglycerate across four rice cultivars (Giza 179, Hassawi rice, Super 300, and Y EGY) under control (**C**) and PEG-induced drought stress (P). (**B**) Root tissue metabolite profiles displaying raw concentrations (left panels) and normalized concentrations (right panels) for benzoin 1TMS, proline 2TMS, galactaric acid 6TMS, leucine 2TMS, trehalose 8TMS, and oxalic acid 2TMS across the four cultivars under both conditions. Box plots indicate median (center line), interquartile range (box edges), minimum and maximum values (whiskers), and individual outliers. Color codes differentiate cultivars: green (Giza 179), cyan (Hassawi rice), yellow (Super 300), magenta (Y EGY), and brown (combined treatment groups). Each metabolite panel presents both absolute concentration and normalized relative abundance, aiding comparison of cultivar-specific drought responses. These metabolites, selected for their high Variable Importance in Projection (VIP) scores, serve as key biomarkers for cultivar differentiation and drought stress adaptation.

Box plot analysis revealed distinct patterns of metabolite accumulation across the four rice cultivars under both control and drought stress conditions, providing detailed insights into cultivar-specific metabolic responses at the individual metabolite level. In leaf tissues (Fig. 6A), succinic acid, a critical TCA cycle intermediate, showed markedly elevated concentrations in Giza 179 under drought stress compared to control conditions, with original concentrations exceeding 2e + 07, while other cultivars exhibited more moderate increases or relatively stable levels. The normalized concentration data further confirmed that Giza 179 accumulated significantly higher relative amounts of succinic acid under stress, indicating enhanced TCA cycle activity and energy metabolism. GABA, a key stress-signaling molecule and metabolite involved in stress tolerance, demonstrated cultivar-specific accumulation patterns, with Y EGY and Super 300 showing pronounced increases under PEG treatment, reaching original concentrations of approximately 4e + 05. At the same time, Giza 179 and Hassawi rice exhibited more controlled accumulation. The normalized concentration data revealed that GABA levels were differentially regulated across cultivars, with some maintaining relatively consistent levels between control and stress conditions. In contrast, others showed dramatic induction, suggesting different regulatory mechanisms for GABA metabolism among the varieties. Citric acid, the entry point of the TCA cycle, displayed the highest accumulation in Giza 179 under stress conditions, with original concentrations reaching approximately 5e + 07, significantly exceeding those observed in other cultivars. This pronounced accumulation, confirmed by normalized concentration analysis, indicates robust activation of the citrate synthase reaction and suggests that Giza 179 maintains high TCA cycle flux under drought stress to support energy demands and biosynthetic processes.

Palmitic acid, a major saturated fatty acid component of membrane lipids, showed substantial increases across all cultivars under drought stress, with Giza 179 again displaying the highest original concentrations (approximately 1e + 07), followed by Hassawi rice and Super 300, while Y EGY showed more moderate accumulation. The normalized concentration data demonstrated that this increase was proportionally significant across all varieties, suggesting a universal response involving membrane lipid remodeling to maintain membrane integrity and fluidity under water deficit conditions. Arbutin, a phenolic glycoside with antioxidant properties, exhibited dramatic cultivar-specific differences, with Super 300 showing exceptionally high original concentrations (exceeding 4000) under stress conditions, while other cultivars, particularly Giza 179 and Hassawi rice, maintained relatively low levels. This striking difference in arbutin accumulation, clearly visible in both original and normalized concentration plots, suggests that Super 300 employs a phenolic compound-based antioxidant strategy that differs fundamentally from the other cultivars. 3-Phosphoglycerate, a key intermediate in glycolysis and photorespiration, showed elevated levels in Giza 179 and Super 300 under stress, with original concentrations reaching approximately 100,000, while Hassawi rice and Y EGY displayed lower accumulation. The normalized concentration analysis confirmed that Giza 179 and Super 300 maintain higher relative levels of this metabolite, potentially reflecting sustained glycolytic activity or enhanced photorespiratory flux as adaptive mechanisms under drought stress.

In root tissues (Fig. 6B), benzoin 1TMS displayed cultivar-specific accumulation patterns with Y EGY showing the highest original concentrations (approximately 40000) under stress conditions, while other cultivars exhibited more moderate levels. The normalized concentration data revealed that benzoin accumulation was particularly pronounced in Y EGY, suggesting a role in root-specific stress responses or secondary metabolism activation in this cultivar. Proline 2TMS, one of the most well-characterized osmolytes and stress markers, showed substantial increases across all cultivars under drought stress, with original concentrations ranging from 50,000 to 110,000, consistent with earlier proline quantification results. However, the box plot analysis revealed important differences in the magnitude and distribution of proline accumulation, with Y EGY and Super 300 showing the highest levels and greater variability, while Giza 179 displayed more moderate but consistent accumulation. The normalized concentration data confirmed that proline induction was proportionally significant in all cultivars, but the extent varied, reflecting different osmotic adjustment strategies. Galactaric acid 6TMS, a sugar acid derivative, exhibited distinct accumulation patterns with Y EGY and Super 300 showing elevated original concentrations (approximately 150000 and 100000, respectively) under stress, while Giza 179 and Hassawi rice maintained lower levels. This differential accumulation, evident in both original and normalized plots, suggests variety-specific differences in sugar metabolism and oxidative pathways in roots under drought conditions.

Leucine 2TMS, a branched-chain amino acid, showed remarkable cultivar-specific responses, with Giza 179 displaying exceptionally high original concentrations (exceeding 2000000) under stress conditions, substantially higher than all other cultivars. This pronounced accumulation, confirmed by normalized concentration analysis, indicates that Giza 179 may utilize leucine and other branched-chain amino acids as alternative respiratory substrates or nitrogen storage compounds under stress, representing a distinctive metabolic strategy. Hassawi rice and Super 300 showed more moderate leucine accumulation, while Y EGY maintained relatively lower levels. Trehalose 8TMS, a non-reducing disaccharide known for its osmoprotective and protein-stabilizing properties, exhibited significant accumulation across cultivars with distinct patterns. Original concentrations showed that Giza 179, Hassawi rice, and Super 300 all accumulated substantial amounts of trehalose under stress (ranging from 1.0e + 07 to 1.5e + 07), while Y EGY showed comparatively lower accumulation. The normalized concentration data revealed that trehalose induction was particularly robust in Hassawi rice and Giza 179, consistent with their superior drought tolerance performance and suggesting that trehalose-mediated protection plays a critical role in their stress adaptation mechanisms. Oxalic acid 2TMS, a small organic acid involved in various metabolic processes and stress responses, displayed variable accumulation patterns with some cultivars showing increases under stress while others maintained relatively stable levels. The original concentration data indicated that Y EGY accumulated higher levels of oxalic acid under stress (approximately 2.5e + 06), while the normalized concentration analysis revealed that relative changes varied considerably among cultivars, suggesting different roles for oxalic acid in cultivar-specific stress responses. The comprehensive box plot analysis of both original and normalized concentrations across these key discriminatory metabolites reveals several important insights into cultivar-specific drought adaptation strategies. First, Giza 179 consistently showed elevated levels of TCA cycle intermediates (succinic acid, citric acid) and energy-related metabolites in leaves, along with exceptionally high leucine and trehalose accumulation in roots, indicating a strategy based on maintaining robust primary metabolism and energy production under stress. Second, Hassawi rice demonstrated more balanced metabolite profiles with strategic accumulation of protective compounds like trehalose without the extreme perturbations observed in other cultivars, consistent with its efficient stress response mechanism. Third, Super 300 exhibited unique phenolic compound accumulation (arbutin) in leaves and moderate but consistent responses in roots, suggesting a hybrid strategy combining antioxidant defense with metabolic adjustment. Fourth, Y EGY displayed the most variable metabolite profiles with extreme accumulation of certain compounds (GABA, proline, benzoin) coupled with lower levels of others, indicating a less coordinated stress response that may contribute to its lower drought tolerance. The comparison between original and normalized concentration data was particularly informative, as it revealed both absolute quantitative differences and relative proportional changes, demonstrating that some metabolites showed universal induction across cultivars (proline, palmitic acid) while others displayed highly cultivar-specific patterns (arbutin, leucine, citric acid). These findings underscore the complexity of metabolic responses to drought stress and highlight how different cultivars employ distinct combinations of metabolic adjustments to cope with water deficit, providing valuable insights for understanding the biochemical basis of drought tolerance and identifying potential biomarkers for breeding programs.

### Metabolic pathway enrichment analysis

**Fig. 7:**
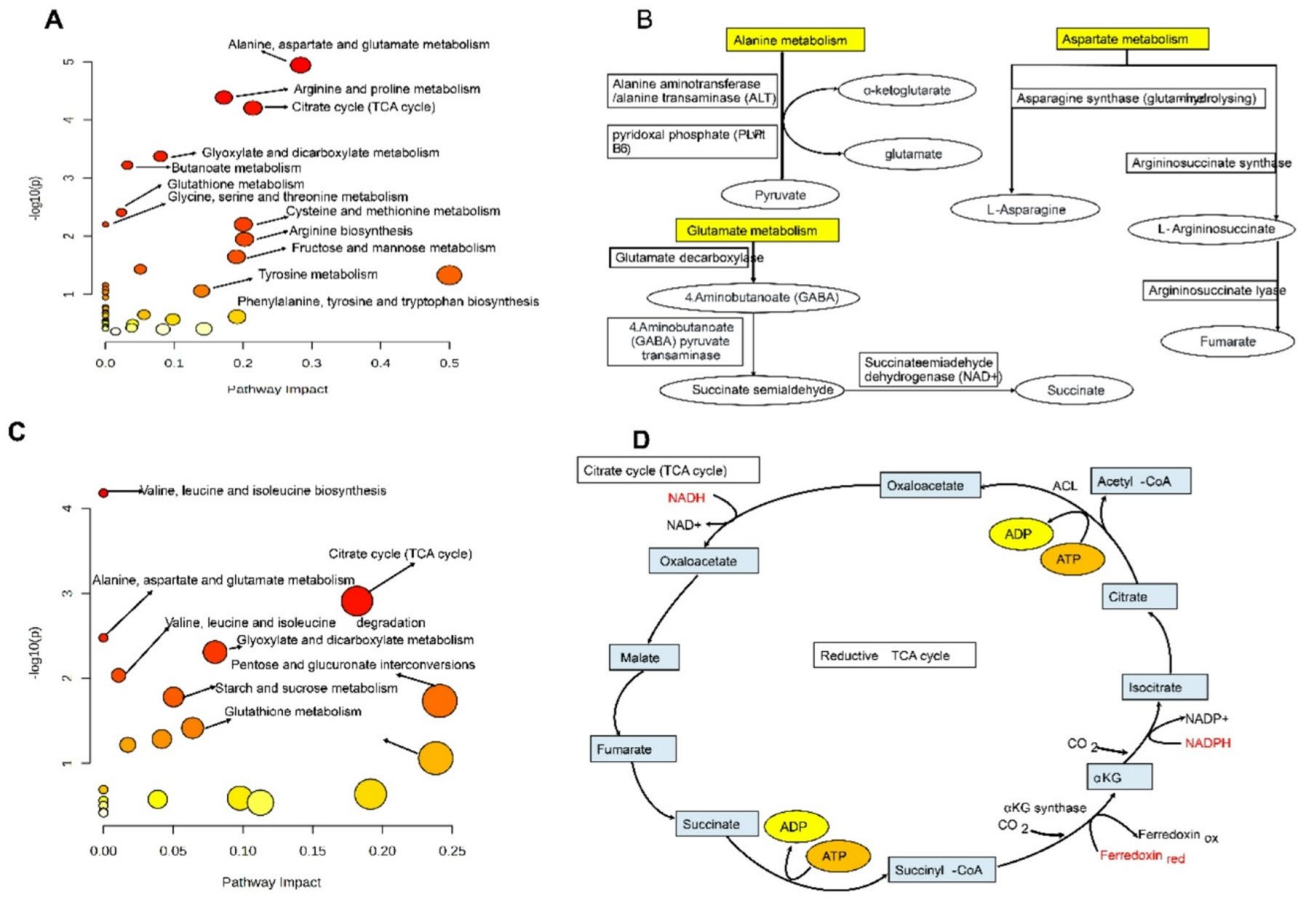
Metabolic pathway enrichment analysis and regulatory mechanisms in rice under drought stress. (**A**) Pathway impact analysis of leaf tissue metabolites highlights enriched metabolic pathways. The bubble plot depicts pathway impact (x-axis) against statistical significance as -log10(p-value) (y-axis). Bubble size correlates with the number of metabolites involved; color gradient reflects significance level (red = highly significant, yellow = less significant). (**B**) Regulatory mechanism of alanine metabolism shows enzymatic conversion via alanine aminotransferase/alanine transaminase (ALT), with pyridoxal phosphate (PLP) as a cofactor, linking α-ketoglutarate, glutamate, and pyruvate. (**C**) Pathway impact analysis of root tissue metabolites reveals enriched pathways under drought stress, using the same bubble plot format. Citrate cycle (TCA cycle) emerges as the most significantly impacted pathway in roots. (**D**) Detailed regulation of the citrate cycle (TCA cycle) in roots illustrates sequential enzymatic reactions, energy molecule production (ATP/ADP), cofactor roles (NADH/NAD+, NADPH/NADP+), electron carriers (ferredoxin), and the reductive TCA branch. Yellow boxes emphasize key pathways; arrows show metabolic flow and enzymatic conversions. Metabolites are in oval/rectangular boxes with cofactors in red text.

Pathway enrichment analysis using MetaboAnalyst (version 5.0) revealed the complex network of metabolic pathways involved in drought stress response in *Oryza sativa* plants at both leaf and root levels. At the leaf level (Fig. 7A), pathway impact analysis demonstrated that several dominant metabolic pathways were significantly enriched under drought conditions, with varying degrees of impact on the overall metabolic network. The pathways exhibiting the highest impact scores included alanine, aspartate and glutamate metabolism, arginine and proline metabolism, and the citrate cycle (TCA cycle), all showing pathway impact values exceeding 0.2 with high statistical significance (-log10(p) > 4). These pathways represent critical nodes in the plant’s metabolic network that are mobilized to cope with water deficit stress. Additional significantly enriched pathways included glyoxylate and dicarboxylate metabolism, butanoate metabolism, glutathione metabolism, glycine, serine, and threonine metabolism, cysteine and methionine metabolism, arginine biosynthesis, fructose and mannose metabolism, and tyrosine metabolism, all demonstrating substantial pathway impact values ranging from 0.1 to 0.3. The bubble plot visualization clearly illustrated that pathways with larger bubble sizes and darker colors (red to orange) represented higher statistical significance and greater metabolic perturbation under drought stress, while smaller yellow bubbles indicated pathways with lower impact but still significant involvement in the stress response. The enrichment of amino acid metabolism pathways, particularly those involving alanine, aspartate, and glutamate, reflects the plant’s strategy to maintain nitrogen balance and produce stress-protective compounds under drought conditions.

The detailed regulatory mechanisms illustrated in Fig. 7B, C, and D provide molecular-level insights into the enzymatic conversions within these critical pathways. In alanine metabolism (Fig. 7B), the pathway demonstrates the conversion of alanine via alanine aminotransferase/alanine transaminase (ALT) enzyme, which catalyzes the reversible transamination reaction between alanine and α-ketoglutarate to produce pyruvate and glutamate, with pyridoxal phosphate (PLR B6) serving as an essential cofactor. This conversion is essential for maintaining cellular homeostasis and connecting amino acid metabolism with central carbon metabolism through pyruvate, which serves as a critical junction between glycolysis and the TCA cycle. In glutamate metabolism (Fig. 7C), the pathway shows glutamate decarboxylase catalyzing the conversion of glutamate to 4-aminobutanoate (GABA), a crucial stress-responsive metabolite that accumulates under various abiotic stresses. The subsequent metabolism involves 4-aminobutanoate (GABA) pyruvate transaminase and succinate semialdehyde dehydrogenase (NAD+), which converts GABA through succinate semialdehyde to succinate, thereby linking GABA metabolism directly to the TCA cycle. In aspartate metabolism (Fig. 7D), asparagine synthase (glutamine-hydrolyzing) catalyzes the conversion of L-aspartate to L-asparagine, while argininosuccinate synthase and argininosuccinate lyase participate in the sequential conversion through L-argininosuccinate to fumarate and succinate, demonstrating the interconnection between amino acid metabolism and the TCA cycle. These enzymatic conversions are essential for maintaining cellular homeostasis, nitrogen assimilation, and energy production under stress conditions.

At the root level (Fig. 7C), several metabolic pathways were significantly enriched, with the citrate cycle (TCA cycle) being the most notably affected pathway, exhibiting the highest impact score (around 0.28) and high statistical significance (-log10(p) > 4). This underscores its vital role in root metabolism during drought stress. Other key pathways enriched in roots included the biosynthesis of valine, leucine, and isoleucine; metabolism of alanine, aspartate, and glutamate; degradation of valine, leucine, and isoleucine; glyoxylate and dicarboxylate metabolism; pentose and glucuronate interconversions; starch and sucrose metabolism; and glutathione metabolism. All of these showed substantial impact values and significance. The importance of the citrate cycle (Fig. 7D) emphasizes its central role in energy production and providing carbon skeletons during drought stress, acting as a hub that links carbohydrate, amino acid, and lipid metabolism. The regulation of this cycle involves a series of enzymatic steps beginning with the condensation of acetyl-CoA and oxaloacetate to produce citrate via ATP-dependent citrate lyase (ACL). It then converts to isocitrate, generating NADPH from NADP+. Further, isocitrate undergoes oxidative decarboxylation to form α-ketoglutarate (αKG), releasing CO₂ and producing NADPH through αKG synthase with ferredoxin serving as an electron carrier (from oxidized to reduced ferredoxin). The cycle continues with succinyl-CoA formation and ATP generation, conversion to succinate with further ATP production, oxidation to fumarate, hydration to malate, and oxidation back to oxaloacetate while generating NADH from NAD+. This completes the cycle. Additionally, the diagram depicts the reductive TCA cycle branch, which functions in reverse and plays a role in biosynthesis and carbon fixation under specific stress conditions.

The enrichment of branched-chain amino acid metabolism (valine, leucine, and isoleucine) in roots suggests active protein turnover and the utilization of amino acids as alternative respiratory substrates when carbohydrate availability is limited under drought conditions. The activation of glyoxylate and dicarboxylate metabolism in both tissues indicates the operation of alternative metabolic routes that bypass certain steps of the TCA cycle, potentially serving as an adaptive mechanism to maintain metabolic flux and regenerate key metabolic intermediates under stress conditions. The significant involvement of glutathione metabolism in both leaf and root tissues underscores the importance of antioxidant defense systems in protecting cells from oxidative damage associated with drought stress. These primary metabolic pathways are essential for plant survival, growth, and development, as amino acids produced serve as building blocks for proteins crucial for structural integrity and enzymatic activity, while the TCA cycle intermediates provide precursors for numerous biosynthetic pathways and energy in the form of reducing equivalents (NADH and NADPH) and ATP. Overall, the pathway enrichment analysis demonstrates that drought stress triggers a coordinated reprogramming of central metabolic pathways in rice plants, with tissue-specific adjustments that reflect the distinct physiological roles of leaves and roots in stress adaptation. The strategic activation of these metabolic pathways facilitates the basic physiological processes required for plant growth, energy production, cellular repair, and reproduction, thereby maintaining plant health and productivity under adverse environmental conditions while optimizing resource allocation between different metabolic networks.

## Discussion

### Distinct physiological reactions to drought stress in different rice cultivars

This study reveals notable differences among four rice cultivars in their drought tolerance mechanisms, with Giza 179 showing the best performance under drought stress. The findings align with prior research indicating that rice drought tolerance is a complex, multigenic trait with considerable genotypic variation^41,42^. The observed 85.2% drought tolerance index for Giza 179 is comparable to that of elite drought-tolerant rice genotypes like IR24 (developed by IRRI) and Vandana, an upland cultivar from India. It both yielded more grains under water-limited conditions and exhibited the highest resistance to drought stress^43^. Fresh weight measurements showed significant reductions in all PEG-treated cultivars, consistent with numerous studies demonstrating that osmotic stress and ion toxicity are the primary mechanisms limiting plant growth under drought conditions^44,45^. However, the differential response patterns observed among cultivars suggest distinct adaptive strategies. Y EGY exhibited the most severe decline (*P* < 0.001), while Giza 179 maintained relatively higher biomass (*P* < 0.01), indicating greater resilience to drought conditions and superior capacity for growth maintenance under water deficit. This biomass retention under stress is a critical trait for yield stability in drought-prone environments and represents a key breeding target for developing climate-resilient rice varieties.

Giza 179 maintained comparatively higher water status, suggesting superior osmoregulation under water deficit, while Y EGY showed the most substantial reduction in RWC (*P*< 0.001). This finding corroborates previous research by Vendruscolo et al^46^., which indicated that RWC is a reliable physiological predictor of drought tolerance, with resistant varieties maintaining higher RWC than susceptible ones. Keeping cellular water balance during drought is essential for photosynthesis, metabolic activities, and crop yield. Notably, Y EGY exhibited the highest increase in proline content, followed by Super 300 and Hassawi rice, while Giza 179 showed a more moderate increase. This presents an intriguing paradox in drought stress biology: the most drought-sensitive cultivar (Y EGY, 52.2% tolerance index) accumulated the most proline, whereas the most tolerant cultivar (Giza 179, 85.2% tolerance index) had only moderate proline levels. This inverse relationship between proline accumulation and drought tolerance, though counterintuitive, has been increasingly documented in recent literature and reflects fundamental differences in stress severity and metabolic efficiency among cultivars^47,48^. The proline paradox can be reconciled through several interconnected mechanistic explanations. First, proline accumulation patterns reflect stress severity rather than tolerance capacity high proline levels often indicate severe cellular damage and metabolic crisis rather than effective adaptation. In tolerant cultivars like Giza 179, moderate proline accumulation suggests controlled, efficient osmoregulation where proline synthesis is precisely regulated to balance osmotic adjustment with growth maintenance. Conversely, excessive proline accumulation in sensitive cultivars represents a metabolic “emergency response” where plants divert substantial carbon and nitrogen resources toward proline synthesis in a desperate attempt to survive extreme cellular dehydration, often at the expense of growth and other essential processes. This interpretation aligns with recent findings showing that constitutive proline overaccumulation can actually impair growth under non-stress conditions and provides no fitness advantage under moderate drought, whereas regulated, moderate accumulation effectively supports stress tolerance. Proline synthesis from glutamate requires ATP and reducing equivalents (NADPH), resources that could otherwise support growth, defense responses, or reproductive development. Giza 179’s moderate proline strategy suggests metabolic efficiency, synthesizing just enough proline for effective osmotic adjustment without depleting energy reserves needed for biomass maintenance (reflected in its 85.2% tolerance index). In contrast, Y EGY’s massive proline accumulation likely represents metabolic inefficiency, where excessive resources are channeled into proline production without proportional benefit for stress tolerance, contributing to the severe growth reductions (52.2% tolerance index) observed under drought. Giza 179’s metabolic profile showed robust activation of the TCA cycle (high citric acid, succinic acid) alongside moderate proline accumulation, indicating that this cultivar maintains energy metabolism to support both stress responses and growth. Y EGY, despite high proline levels, showed weaker overall metabolic reprogramming and fewer significantly altered metabolites, suggesting that proline accumulation occurred in isolation without the coordinated metabolic adjustments necessary for effective stress tolerance. This supports the interpretation that Y EGY’s high proline represents a symptom of stress damage rather than an adaptive response—the plant produces proline reactively in response to severe cellular dysfunction but lacks the integrated metabolic capacity to translate this accumulation into functional tolerance.

Giza 179’s moderate proline accumulation, coupled with maintained biomass, high RWC, and robust metabolic activity, indicates effective stress management with minimal cellular damage. Y EGY’s extreme proline accumulation, occurring alongside severe biomass loss, substantial RWC decline, and limited metabolic reprogramming, signals catastrophic cellular stress where proline synthesis represents a futile rescue attempt rather than successful adaptation. This interpretation has important breeding implications: selection should focus on cultivars exhibiting moderate, regulated proline responses integrated within broader metabolic stress management strategies, rather than targeting maximum proline accumulation capacity, which may actually indicate stress sensitivity rather than tolerance^49,50^.

### Metabolomic insights into drought tolerance mechanisms

GC-MS metabolomic analysis showed that different plant tissues respond uniquely to drought stress, as evidenced by distinct clustering patterns in PCA analysis. This suggests that metabolic reprogramming varies across tissues. Such tissue-specific responses are consistent with multiple studies indicating that roots and shoots adopt fundamentally different metabolic strategies during abiotic stress adaptation^13,50–52^. The metabolic diversity patterns observed show that roots shared only three conserved metabolites across cultivars, whereas leaves shared four. This suggests that root tissues have greater metabolic flexibility and cultivar-specific adaptation strategies. This is biologically important because roots are the first organ exposed to water stress, acting as the initial site for stress detection, signal transduction, and early adaptive responses^53–56^. The twice-as-high metabolic diversity in roots compared to leaves indicates that drought tolerance specific to cultivars is mainly influenced by the roots’ ability to reprogram their metabolism, rather than by responses at the leaf level. This tissue-specific metabolic differentiation has also been observed in other stress studies, where roots demonstrated greater metabolic flexibility and more significant cultivar-dependent reactions to drought stress^57^.

### Cultivar-specific metabolic signatures and tolerance mechanisms

The metabolomic analysis of leaf tissues revealed distinct biochemical signatures for each cultivar, providing insights into their specific tolerance mechanisms. Giza 179 exhibited increased levels of succinic acid, GABA, L-methionine, and palmitic acid under drought stress compared to non-stressed conditions. These metabolites are vital and interconnected in drought adaptation. Succinic acid, a key part of the TCA cycle, aids respiration and energy production during stress and acts as a precursor for protective compounds^58^. GABA acts as a signaling molecule that controls stomatal closure, boosts antioxidant enzymes, and helps maintain ion balance^59^. L-methionine is a precursor for S-adenosylmethionine (SAM) creation, essential for polyamine synthesis and methylation reactions that aid stress adaptation^60^. Palmitic acid helps stabilize membranes and reinforce the cuticle, decreasing water loss by strengthening hydrophobic barriers^17^.

The coordinated accumulation of these metabolites in Giza 179 reflects a comprehensive metabolic strategy to address drought stress. It involves energy metabolism (succinic acid), signaling and ROS scavenging (GABA), methylation and polyamine synthesis (L-methionine), and membrane protection (palmitic acid). This multifaceted approach explains Giza 179’s improved drought tolerance and provides a metabolic signature that could be utilized in breeding programs. Super 300 tends to accumulate succinic acid, mannose-6-phosphate, palmitic acid, and citric acid in leaf tissues under drought conditions. The presence of mannose-6-phosphate, a key component of the pentose phosphate cycle and glycolysis, indicates activation of alternative carbohydrate pathways capable of generating NADPH for antioxidant defense^61^. Citric acid accumulation reflects TCA cycle modulation and potential pH regulation in cellular compartments^58^. The shared accumulation of succinic acid and palmitic acid between Giza 179 and Super 300 suggests common elements in their stress response strategies, consistent with their relatively closer tolerance indices (112.6% vs. 80.9%). Hassawi rice demonstrated preferential accumulation of methyl galactosidase, L-arginine, 2-deoxyuridine, arbutin, and mannose isomer in leaf tissues under drought stress. L-arginine serves as a precursor for polyamine and nitric oxide biosynthesis, both critical signaling molecules in stress responses. Arbutin, a glycosylated hydroquinone, functions as an antioxidant and may contribute to ROS detoxification. The unique metabolic profile of Hassawi rice, despite its intermediate tolerance index (93.3%), suggests that this cultivar employs distinct biochemical pathways compared to the other varieties, potentially involving nucleotide metabolism (2-deoxyuridine) and specialized secondary metabolite production. Y EGY demonstrated preferential accumulation of glucopyranose, 3-phosphoglycerate, and arbutin in leaf tissues under drought stress. The elevation of 3-phosphoglycerate, a primary photosynthetic product, may reflect disrupted carbon assimilation and metabolic bottlenecks in downstream pathways. The limited diversity of significantly accumulated metabolites in Y EGY compared to other cultivars suggests a less comprehensive or less effective metabolic response, consistent with its lowest tolerance index (69.1%).

### Root metabolic responses and their significance

Root tissue analysis revealed more distinct and cultivar-specific metabolic changes compared to leaves, highlighting the vital role of root metabolism in drought adaptation. Under drought stress, Giza 179 exhibited notably higher levels of leucine, proline, trehalose, and oxalic acid in its roots. The accumulation of trehalose is especially important because this non-reducing disaccharide serves as a signalling molecule that regulates carbon distribution and stress-related gene expression, in addition to functioning as an osmoprotectant^29,31^. The most drought-tolerant cultivar’s roots have higher trehalose levels, suggesting that root-localized trehalose metabolism may play a significant role in rice’s resilience to drought. The accumulation of branched-chain amino acids (leucine) in Giza 179 roots indicates enhanced protein synthesis capacity and nitrogen metabolism under stress. Branched-chain amino acids serve as nitrogen storage compounds, alternative respiratory substrates, and precursors for specialized metabolites^62^. Oxalic acid accumulation may assist with metal chelation, pH regulation, and calcium signaling in root tissues. The coordinated increase of these diverse metabolites in Giza 179 roots reflects complex metabolic reprogramming that supports root function during water deficit conditions. Hassawi rice showed significantly higher levels of leucine, citric acid, sucrose, and ribose isomer under drought stress in roots. The accumulation of sucrose, the primary sugar used for photosynthetic transport, in roots indicates continuous carbon allocation to root tissues even under stress and vigorous phloem unloading. This sustained root carbon supply may help maintain root growth and water uptake. Ribose accumulation indicates active nucleotide metabolism and possibly improved capacity for ATP regeneration, which is vital for energy-dependent stress responses^51^.

Y EGY showed significantly higher levels of citric acid, benzoin, and D-mannitol in roots under drought stress. While increased citric acid and mannitol are common stress responses—related to the TCA cycle and osmotic regulation—benzoin, an aromatic compound, suggests activation of specialized phenylpropanoid pathways. The relatively limited metabolic response in Y EGY roots, compared to tolerant cultivars like Giza 179, indicates that Y EGY’s ability to adapt metabolically is insufficient for effective drought resistance. In contrast, Super 300 showed elevated levels of citric acid, benzoin, isocitrate, and galactaric acid in roots during drought. The concurrent rise in citric acid and isocitrate indicates active TCA cycle processes and possible anaplerotic reactions replenishing cycle intermediates used in biosynthesis. The detection of galactaric acid suggests active cell wall metabolism, possibly involving cell wall remodeling to enhance mechanical strength or reduce water loss via the apoplast.

### Variable significance in biomarker identification and projection analysis

The identification of 40 metabolites with VIP scores exceeding 1.0 and 10 metabolites with VIP values over 1.5 offers a refined set of biomarkers for assessing rice drought resistance. The metabolites most capable of distinguishing between the four cultivars’ unique stress responses were selected based on a VIP > 1.5 threshold. This biomarker strategy has proven effective in other crop species, where metabolite-based selection has accelerated breeding programs aimed at improving stress tolerance^63^. The complete metabolomic dataset included 114 polar metabolites from leaf tissues and 97 from root systems, showing the significant metabolic complexity captured through GC-MS analysis. The higher number of metabolites found in leaves compared to roots likely reflects the greater structural and metabolic diversity of photosynthetic tissues, which support complex networks of primary and secondary metabolism. However, the higher discriminatory power of root metabolites for differentiating cultivars (as shown by PCA variance partitioning) indicates that root-specific metabolites serve as more reliable indicators of drought tolerance capacity.

### Integration of physiological and metabolomic data

The integration of morpho-physiological parameters with comprehensive metabolomic profiling offers a holistic view of how rice tolerates drought, revealing the underlying mechanisms. The strong correlation between stress tolerance indices and specific metabolic signatures supports the effectiveness of the metabolomic approach for cultivar selection and breeding. Giza 179 outperforms others across various metrics, achieving the highest tolerance index, maintaining greater biomass and RWC, exhibiting moderate proline accumulation, and displaying unique metabolic signatures in both leaves and roots, indicating it has evolved or been selected for an integrated stress response. This integrated response sharply contrasts with Y EGY’s strategy, which shows excessive proline accumulation but significant growth reductions and limited metabolic diversity. Y EGY’s pattern suggests a “survival mode” focused on immediate cellular protection overgrowth, likely adapted to transient, severe droughts. Volcano plot analysis confirms this, showing Y EGY has fewer significantly altered metabolites and smaller fold-changes than Giza 179, indicating weaker metabolic reprogramming. The intermediate cultivars, Hassawi rice and Super 300, show varying metabolic flexibility and stress adaptation. Hassawi rice’s moderate tolerance involves the strategic upregulation of metabolites such as trehalose in leaves and sucrose in roots, indicating efficient carbon use and osmotic balance. Super 300’s intermediate performance reflects a balanced metabolic response, sharing some features with Giza 179, like increased succinic and palmitic acids, but with less extensive metabolic reprogramming.

### Study limitations, methodological considerations, breeding implications, and future directions

This study offers valuable insights into cultivar-specific drought responses but has limitations. PEG-6000-induced osmotic stress, while controlled and reproducible, doesn’t fully mimic soil drought conditions, lacking features like gradual water deficit, mechanical impedance, and recovery assessment. It creates uniform stress across roots, unlike the heterogeneous soil drought affecting root architecture. Our focus on seedlings captures early responses but may not predict later-stage performance during reproductive phases, where drought impacts yield most. Seedling tolerance mechanisms may differ from those during flowering and grain filling, so seedling responses don’t always correlate with field performance. Nonetheless, seedling screening remains useful for initial evaluation and understanding metabolic responses. Our metabolomic profiling identified biomarkers like trehalose, citric acid, and arbutin associated with tolerance, but these are correlative, not causal. Functional validation, including genetic studies, metabolite applications, and field trials, is needed to confirm their roles and predictive value. Overall, observed metabolite changes may reflect downstream effects rather than direct tolerance mechanisms.

This study’s tissue-specific metabolic responses inform breeding strategies for drought-tolerant rice. Super 300 shows restrained root responses, unlike Y EGY’s disruption, indicating targeted root metabolic adjustments as a more energy-efficient adaptation. Breeding should prioritize root metabolic stability and responses over metabolite levels. Root metabolites can help identify tolerant cultivars, making root-based phenotyping useful for early selection alongside traditional traits. Marker-assisted selection can target genes controlling root trehalose, leucine, and TCA cycle traits linked to drought performance. Cultivar-specific metabolic signatures reveal targets for precision breeding: Giza 179’s TCA cycle stability can be introgressed into high-yield varieties, and Hassawi’s trehalose osmoprotection offers an alternative stress tolerance path. Combining these mechanisms through crossing could produce superior varieties with multiple beneficial traits. Future research should focus on validating biomarkers in field conditions across drought scenarios and growth stages to confirm their usefulness for breeding. Functional validation through genetic manipulation and metabolite application would establish causality with drought tolerance. Combining metabolomics with transcriptomics and proteomics would elucidate regulatory networks and enzyme activities involved in drought adaptation. Expanding studies to diverse rice genotypes and conducting GWAS could identify genetic variants linked to beneficial metabolic responses, aiding the development of climate-resilient rice for global food security.

## Conclusions

Our integrated morpho-physiological and metabolomic analysis shows that rice cultivars use fundamentally different molecular strategies for drought adaptation, with significant implications for global food security. Giza 179 stands out as an elite drought-tolerant genotype through a previously unrecognized integrated stress response mechanism, indicating greater resilience to drought, sustained growth, and metabolic efficiency, achieving 85.2% stress tolerance while avoiding the energetic costs seen in other cultivars. Using comprehensive GC-MS metabolomics, we found tissue-specific metabolic reprogramming patterns. The identification of 10 high-impact metabolic biomarkers (VIP > 1.6) offers molecular targets for next-generation crop improvement. Additionally, our demonstration that effective drought tolerance results from coordinated multi-pathway networks rather than single-gene effects challenges existing breeding paradigms. These findings establish a precision phenotyping framework that shifts stress biology from descriptive to predictive science, providing actionable strategies for developing climate-resilient crops capable of feeding growing populations on increasingly marginal agricultural lands.

## Supplementary Information

Below is the link to the electronic supplementary material.

Supplementary Material 1

Supplementary Material 2

## Data Availability

All data generated or analyzed during this study are included in this published article. Raw GC-MS data files and processed metabolite concentration matrices have been deposited in the MetaboLights repository ( [https://www.ebi.ac.uk/metabolights/editor/study/REQ20260206216897](https:/www.ebi.ac.uk/metabolights/editor/study/REQ20260206216897) ). Also, Raw GC-MS data files were added as supplementary material (Tables S1 and S2). The peak intensity filtering criteria and data preprocessing parameters are documented in the repository metadata. The data, metadata, and repository are available from the corresponding author, Sobhi F. Lamlom, upon reasonable request.
